# MALDI-TOF mass spectrometry as a potential tool for *Trichomonas vaginalis* identification

**DOI:** 10.1186/s12879-016-1594-z

**Published:** 2016-06-10

**Authors:** Adriana Calderaro, Maddalena Piergianni, Sara Montecchini, Mirko Buttrini, Giovanna Piccolo, Sabina Rossi, Maria Cristina Arcangeletti, Maria Cristina Medici, Carlo Chezzi, Flora De Conto

**Affiliations:** Unit of Microbiology and Virology, Department of Clinical and Experimental Medicine, University of Parma, Viale A. Gramsci, 14-43126 Parma, Italy

**Keywords:** *Trichomonas vaginalis*, MALDI-TOF MS, Proteic profile, Parameter setting

## Abstract

**Background:**

*Trichomonas vaginalis* is a flagellated protozoan causing trichomoniasis, a sexually transmitted human infection, with around 276.4 million new cases estimated by World Health Organization. Culture is the gold standard method for the diagnosis of *T. vaginalis* infection. Recently, immunochromatographic assays as well as PCR assays for the detection of *T. vaginalis* antigen or DNA, respectively, have been also available. Although the well-known genome sequence of *T. vaginalis* has made possible the application of proteomic studies, few data are available about the overall proteomic expression profiling of *T. vaginalis*. The aim of this study was to investigate the potential application of MALDI-TOF MS as a new tool for the identification of *T. vaginalis*.

**Methods:**

Twenty-one isolates were analysed by MALDI-TOF MS after the creation of a Main Spectrum Profile (MSP) from a *T. vaginalis* reference strain (G3) and its subsequent supplementation in the Bruker Daltonics database, not including any profile of protozoa. This was achieved after the development of a new identification method created by modifying the range setting (6–10 kDa) for the MALDI-TOF MS analysis in order to exclude the overlapping of peaks derived from the culture media used in this study.

**Results:**

Two MSP reference spectra were created in 2 different range: 3–15 kDa (standard range setting) and 6–10 kDa (new range setting). Both MSP spectra were deposited in the MALDI BioTyper database for further identification of additional *T. vaginalis* strains. All the 21 strains analysed in this study were correctly identified by using the new identification method.

**Conclusions:**

In this study it was demonstrated that changes in the MALDI-TOF MS standard parameters usually used to identify bacteria and fungi allowed the identification of the protozoan *T. vaginalis*. This study shows the usefulness of MALDI-TOF MS in the reliable identification of microorganism grown on complex liquid media such as the protozoan *T. vaginalis*, on the basis of the proteic profile and not on the basis of single markers, by using a “new range setting” different from that developed for bacteria and fungi.

## Background

*Trichomonas vaginalis* is a flagellated protozoan of the urogenital tract belonging to the order *Trichomonadida* [1] and it is the aetiologic agent of trichomoniasis [2], the most common non-viral sexually transmitted infection (STI) worldwide [3]. As reported by the World Health Organization (WHO), the 2008 global estimate of the number of new cases for trichomoniasis is 11.2 % higher than the estimate for 2005 (276.4 million *versus* 248.5 million) [4].

The main pathological manifestations of *T. vaginalis* infection in women are abdominal pain, itching, and the presence of a foul-smelling discharge with abundant leukocytes, while in men the infection is mostly asymptomatic, although it can sometimes lead to urethritis, prostatitis, and epididymitis [2, 5].

The visualisation of the morphostructural characteristics of the motile parasites by microscopic examination of wet mounts (sensitivity 51–65 %) from vaginal and urethral secretions is the most practical and rapid but relatively insensitive method used for the diagnosis of trichomoniasis [6]. Direct immunofluorescent antibody staining is more sensitive than wet mounts but technically complex. Culture (sensitivity 75–96 %, specificity 100 %) of the parasite is the gold standard for the diagnosis of trichomoniasis, but it is not widely used and the results are not available for 3 to 7 days [6]. Recently, immunochromatographic assays (sensitivity 82–95 %, specificity 97–100 %) for the detection of specific antigens of *T. vaginalis*, as well as PCR assays (sensitivity and specificity 95–100 %) for the detection of *T. vaginalis* DNA have been also available [2, 6–8].

The *T. vaginalis* genome sequencing was completed in 2007 [9, 10]. The parasite genome is organized in six chromosomes and the number of protein-coding genes is estimated at about 60,000 [10]. The availability of *T. vaginalis* genomic sequences makes possible and practical the application of proteomic methods to the study of global patterns of gene expression. Several laboratories have contributed to understanding the protein expression of *T. vaginalis* [10]. However, these studies are focused on specific proteins, and little is known about the overall profile of this parasite [10].

Matrix-assisted laser desorption/ionization time-of-flight mass spectrometry (MALDI-TOF MS) is widely employed to determine the mass of peptides and proteins [11]. Key aspect of whole-cell MALDI-TOF MS is the rapid generation and comparison of mass spectra. The peaks of these mass spectra represent abundant cellular proteins [12]. The quality of these spectra was improved particularly by the optimization of sample preparation procedure and matrix composition [13]. This technology has been adopted for the rapid identification of bacterial and fungal isolates in the clinical microbiology laboratory where it has replaced traditional identification methods [11, 14–17].

In human parasitology, MALDI-TOF MS has had limited application, such as the detection of malarial hemozoin in blood [18], parasites identification [19–21] or detection of their biomarkers by MALDI-TOF MS (eg. [22–25]). Recently, MALDI-TOF MS was applied for the first time to the differentiation of *E. histolytica* and *E. dispar* strains isolated from clinical samples [26].

The aim of this study was the development of a MALDI-TOF MS identification method for microorganisms different from bacteria and fungi that grow on complex liquid complex media. In this study for the first time the potential application of MALDI-TOF MS as a new tool for the reliable identification of *T. vaginalis* was investigated. This was done by the implementation of the commercial database (version 3.1.66) of the MALDI-TOF mass spectrometer currently used in our laboratory, with the spectrum of a *T. vaginalis* reference strain after creation of a specific proteic reference profile.

## Methods

### Strains, samples and culture procedures

Trophozoites of *T. vaginalis* strain G3 [27] (kindly provided by Prof. Pier Luigi Fiori, Department of Biomedical Sciences, University of Sassari, Italy) were axenically cultivated at 37 °C for at least 3 days in “Trypticase-yeast extract maltose” (TYM) medium pH 6.6, supplemented with fetal bovine serum (final concentration 10 %) inactivated at 56 °C for 30 min [28].

Twenty-one *T. vaginalis* strains belonging to our collection (Tv1-Tv21) were isolated from clinical samples (19 vaginal swabs, 1 urine, and 1 urethral swab) during a 10-year period (from 2005 to 2014). For the MALDI-TOF MS analysis the 21 strains were cultivated in “*Trichomonas* medium N.2” (Oxoid, Perth, UK) at 37 °C for at least 3 days and in any case until the elimination of bacteria and yeasts derived from the microbiota of the host. All the 22 cultivated strains (the G3 reference strain and 21 clinical strains) were analysed by MALDI-TOF MS when the trophozoites concentration was 10^6^/ml.

MALDI-TOF MS was also performed directly on a urine sample (N. 20), belonging to a patient with trichomoniasis, sent to our laboratory at room temperature and stored at 4 °C for 5 h before the analysis. Subsequently, a *T. vaginalis* strain (Tv20) was isolated from this sample. Briefly, a 15 ml aliquot of the sample N. 20 was centrifuged and the obtained pellet was re-suspended in 1.5 ml of sterile *Trichomonas* medium N.2. The strain Tv20 was isolated by adding a 2 ml aliquot of the urine sample to 2 ml of *Trichomonas* medium N.2 and cultivated as described above.

Three experimentally seeded samples with the *T. vaginalis* strain (Tv15) were prepared as follows: 1 vaginal swab, 1 urethral swab and 1 urine sample collected in Uriswab™ (Urine Collection Transport & Preservation System, COPAN, Italy), subjected to the diagnosis of infections by microbes other than *T. vaginalis*, all negatives for *T. vaginalis* and other pathogenic microbes, were moistened in a volume of 500 μl of Tv15 at a concentration of 10^6^ trophozoites/ml and subsequently put in 500 μl of double-distilled sterile water to release the absorbed trophozoites. The suspensions obtained (< 10^6^ trophozoites/ml) were subjected to MALDI-TOF MS analysis as described below.

The samples analysed in this study were sent to the University Hospital of Parma for routine diagnostic purposes, and the laboratory diagnosis results were reported in the medical records of the patients as answer to a clinical suspicion; ethical approval at the University Hospital of Parma is required only in cases in which the clinical samples are to be used for applications other than diagnosis. Thus, for this study the ethics committee approval was unnecessary in accordance with local legislation and guidelines.

### Sample preparation for MALDI-TOF MS analysis

Aliquots of 1.5 ml obtained from *T. vaginalis* reference strain G3, 21 isolates, and the urine sample N.20, and aliquots of 0.5 ml obtained from the 3 experimentally seeded samples were subjected to MALDI-TOF MS analysis after protein extraction in ethanol/formic acid, as previously described [15–17] with some modifications. Briefly, each aliquot was centrifuged at 1800 × *g* for 10 min and the supernatant was discarded. The pellet obtained was subjected to two subsequent washing steps in PBS in 1 ml and in 0.2 ml respectively, both followed by centrifugation at 2500 × *g* for 5 min. The obtained pellet was suspended in distilled water and in absolute ethanol in a 1:3 ratio. The suspension was centrifuged at 13,000 × *g* for 10 min and the supernatant was discarded. After 40 min of air-drying to evaporate the ethanol residue, 30 μl of 70 % formic acid and 30 μl of acetonitrile were added and the suspension was vortexed for 1 min and centrifuged at 13,000 × *g* for 2 min. Then, 1 μl of the supernatant was transferred to a polished steel MSP-96 target plate (Bruker Daltonics, Germany) and allowed to dry at room temperature before being overlaid with 1 μl of a satured α-cyano-4-hydroxy-cinnamic acid (HCCA) matrix solution (Bruker Daltonics).

In each session, TYM axenic medium and/or *Trichomonas* medium N.2, subjected to the same protein extraction procedure, were used as controls accordingly to the strains tested. In each experiment, the “Bacterial Test Standard” (Bruker Daltonics) for calibration was used according to the manufacturer’s instructions.

### Spectra acquisition by MALDI-TOF MS

Proteic extracts were analysed by MicroFlex LT mass spectrometer (Bruker Daltonics, supplied by Becton Dickinson, Italy); spectra were acquired in positive linear mode, laser frequency 60 Hz, ion source voltage 20 kV, mass range 2–20 kDa, in manual mode with overall 400 laser-shot by 100 shot steps. Each shot step was made in different points of the well with a laser intensity of 30 % for each single shot step.

For all the reference and clinical strains tested at least 2 independent experiments from 2 independent cultures on 2 different days were run (inter-assay reproducibility) and 8 replicates/run (intra-assay reproducibility) were analysed in order to ensure the reproducibility of the results obtained.

In order to minimize the variability associated with technical or biological parameters, the experiments were performed under controlled cultivation and sample preparation conditions or technical configurations, assuring a high repeatability and reproducibility among experiments.

### Database creation and identification

MALDI BioTyper database was implemented with the Main Spectrum Profile (MSP) of the *T. vaginalis* reference strain G3 created as described below. The raw spectra acquired by MALDI-TOF MS were analysed by FlexAnalysis software to carry out “Smoothing” and “Baseline” and to delete spectra with an overall intensity <10^4^ arbitrary units and spectra fully different from the others. Ten spectra of *T. vaginalis* strain G3 were uploaded in MALDI BioTyper software (Version 3.1, Bruker Daltonics).

Two different MSP-Spectra were created by the automated function of BioTyper software (BioTyper MSP Creation Standard Methods) in two range settings respectively: 6–10 kDa (new parameter used in this study) and 3–15 kDa (default parameter). The new range setting (6–10 kDa) was established after analysis by Flex Analysis software of the spectra obtained for *T. vaginalis* G3, TYM axenic medium, and *Trichomonas* medium N.2 in 3 independent experiments.

All the spectra obtained for the 21 isolated strains were subjected to identification by Biotyper software by both the new and the standard range setting. At the time of the analysis, the database library included 5675 spectra and *T. vaginalis* G3 spectrum. Parasitic spectra were not available in the commercially database library used. Results were expressed as identification scores varying from 0 to 3. According to the manufacturer’s instructions validated for bacteria and fungi and to what reported also for other parasites [20], a score value ≥2 indicates a species level identification; a score value 1.7-2 indicates a genus level identification; and a score value <1.7 indicates an unreliable identification.

### Detection limit of MALDI-TOF MS

In order to evaluate the detection limit of the analysis by MALDI-TOF MS, serial ten-fold dilutions (from 10^6^ to 10 trophozoites/ml) of Tv15 trophozoites were mixed with an equal volume (1.5 ml) of sterile PBS without Ca^2+^ and Mg^2+^. Subsequently, 1 ml of each dilution was centrifuged at 1800 × *g* for 10 min and the pellet obtained was subjected to the same protein extraction procedure described above.

## Results

**Fig. 1:**
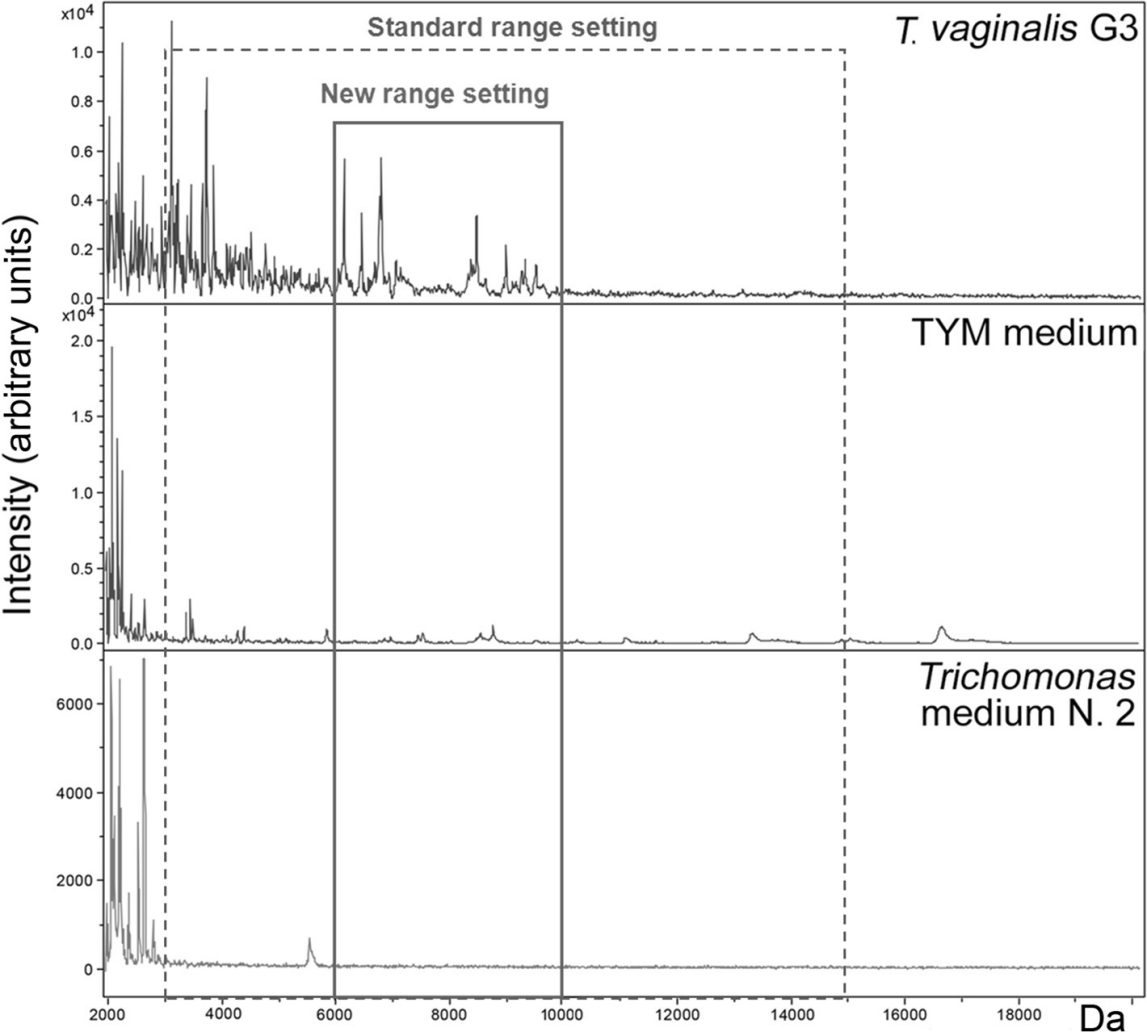
Proteic profiles of the *T. vaginalis* reference strain G3 and of the culture media. Spectra obtained for *T. vaginalis* reference strain G3 and for the media “Trypticase-yeast extract maltose (TYM)” and “*Trichomonas* medium N. 2”, respectively, by MALDI-TOF MS analysis. The box with dashed line highlights the 3 spectra in the molecular range 3–15 kDa; the box with continuous line highlights the 3 spectra in the molecular range 6–10 kDa

The spectra obtained with all the replicates of *T. vaginalis* reference strain G3, acquired in two independent experiments, matched none of the existing spectra profiles in the database (score value <1.2). The spectra of *T. vaginalis* strain G3 and the spectra of the culture medium (TYM axenic medium or of *Trichomonas* medium N. 2) acquired by MALDI-TOF MS and analysed by FlexAnalysis software in the range 2–6 kDa showed common peaks. The same spectra analysed in the range 6–10 kDa did not show common peaks. The spectra of *T. vaginalis* strain G3 analysed in the range 10–20 kDa did not show any peaks (Fig. 1).

Two MSP reference spectra were created in 2 different range: 3–15 kDa (standard range setting) and 6–10 kDa (new range setting).

**Fig. 2:**
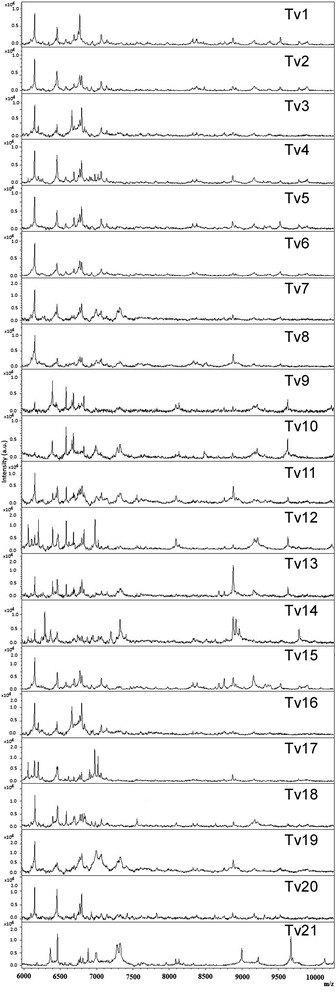
Proteic profiles of the 21 *T. vaginalis* isolates analysed in this study. Spectra obtained with the 21 *T. vaginalis* isolates by MALDI-TOF MS analysis in the molecular range 6–10 kDa

The 2 MSP reference spectra of *T. vaginalis* obtained in the new range setting (6–10 kDa) and in the standard range setting (3–15 kDa), respectively, were both deposited in the MALDI BioTyper database for further identification of additional *T. vaginalis* strains. These reference profiles proved to be reproducible in a second independent experiment, performed after the database implementation, when a correct identification was found for all the replicates of the strain (score of the best match >2.4). In the range setting 6–10 kDa, all the 21 isolates were identified as *T. vaginalis*, 12 (Tv1-Tv8, Tv17-Tv20) with a score value ≥ 2.0 for each of the replicates and 9 (Tv9-Tv16, and Tv21) with a score value comprised between 1.7 and 2.0. In the standard range setting (3–15 kDa), 6 of the 21 strains (Tv1-Tv6) were identified as *T. vaginalis* with a score value comprised between 1.7 and 2.0 and the remaining 15 isolates (Tv7-Tv21) were not identified (Table 1). The spectra obtained for the 21 isolates in the range setting 6–10 kDa were shown in Fig. 2.

**Table 1:** Compared results by MALDI-TOF MS analysis using the new and the standard identification methods

	MALDI-TOF MS analysis			
	New identification method		Standard identification method	
	Range setting (6–10 kDa)		Range setting (3–15 kDa)	
Clinical isolates	Identification	Score	Identification	Score
Tv1^a^	*T. vaginalis* G3	2.0	*T. vaginalis* G3	1.9
Tv2^a^	*T. vaginalis* G3	2.2	*T. vaginalis* G3	1.9
Tv3^a^	*T. vaginalis* G3	2.1	*T. vaginalis* G3	1.7
Tv4^a^	*T. vaginalis* G3	2.1	*T. vaginalis* G3	1.7
Tv5^a^	*T. vaginalis* G3	2.3	*T. vaginalis* G3	1.7
Tv6^a^	*T. vaginalis* G3	2.2	*T. vaginalis* G3	1.7
Tv7^a^	*T. vaginalis* G3	2.2	No identification	NA
Tv8^a^	*T. vaginalis* G3	2.1	No identification	NA
Tv9^a^	*T. vaginalis* G3	1.7	No identification	NA
Tv10^a^	*T. vaginalis* G3	1.7	No identification	NA
Tv11^a^	*T. vaginalis* G3	1.7	No identification	NA
Tv12^a^	*T. vaginalis* G3	1.9	No identification	NA
Tv13^a^	*T. vaginalis* G3	1.8	No identification	NA
Tv14^a^	*T. vaginalis* G3	1.7	No identification	NA
Tv15^a^	*T. vaginalis* G3	1.7	No identification	NA
Tv16^a^	*T. vaginalis* G3	1.7	No identification	NA
Tv17^a^	*T. vaginalis* G3	2.0	No identification	NA
Tv18^a^	*T. vaginalis* G3	2.0	No identification	NA
Tv19^a^	*T. vaginalis* G3	2.0	No identification	NA
Tv20^b^	*T. vaginalis* G3	2.0	No identification	NA
Tv21^c^	*T. vaginalis* G3	1.7	No identification	NA
Samples				
Urine N. 20	No identification	NA	No identification	NA
Vaginal swab^d^	No identification	NA	No identification	NA
Urethral swab^d^	No identification	NA	No identification	NA
Urine swab^d^	No identification	NA	No identification	NA

Tv *Trichomonas vaginalis*, *NA* not applicable

^a^isolated from vaginal swab

^b^isolated from urine

^c^isolated from urethral swab

^d^experimentally seeded sample with Tv15

No identification was obtained when the MALDI-TOF MS was applied both on the pellet obtained from the urine sample N. 20 (score value < 1.4) and on the 3 samples experimentally seeded (1 vaginal swab, 1 urethral swab and 1 urine sample collected in Uriswab™) with the strain Tv15 (score value <1.7).

In this study, MALDI-TOF MS for the identification of *T. vaginalis* showed a detection limit of 10^5^ trophozoites/ml.

## Discussion

MALDI-TOF MS has revolutionised the diagnostic work-flow in bacteriology and mycology replacing traditional identification methods [11]. Thus, now it represents one of the most used techniques for the identification of pathogenic microorganisms [13, 19, 20]. However, in parasitology, MALDI-TOF MS had limited applications such as general parasitic proteome studies and the characterisation of specific biomarkers. As a matter of fact, its diagnostic use in parasitology has remained limited [20], only being applied to *Leishmania* sp. identification [19, 21], to the discrimination of microsporidian isolates grown on cell cultures [24], and to the typing of *Blastocystis* sp. from axenic cultures [20]. Recently, our group had demonstrated the usefulness of MALDI-TOF MS for the differentiation of *Entamoeba histolytica* and *E. dispar* [26].

MALDI-TOF MS was previously used for the assessment of the first reference proteome map of *T. vaginalis* [10] and, to our knowledge, the present study is the first reporting the application of MALDI-TOF MS to the identification of *T. vaginalis*.

In this study MALDI-TOF MS was applied to identify 21 *T. vaginalis* isolates after the creation of a specific proteic reference profile by using the *T. vaginalis* reference strain G3.

The proteic profile of the *T. vaginalis* reference strain G3 was found to be original, matching none of the existing profile in the Bruker Daltonics database even if some peaks overlapped those present in the spectra of the culture media used in this study. The new identification method applied in this study, based on the creation of a MSP-spectrum in the 6–10 kDa mass range to identify unknown spectra, allowed to overcome these overlapping. Although, as already reported, metabolites, pigment and the other materials present in a culture medium may interfere with the crystallisation process and lead to a lower score values [20], in the selected mass-range (6–10 kDa) the components of the both media used did not affect the identification of *T. vaginalis.* In fact, this new method has proved to be necessary because in 15 cases allowed the correct identification of the protozoan not otherwise achievable by the standard mode, and in 6 cases it allowed the identification with a better score as compared to the standard mode. The correct identification of *T. vaginalis* isolates by means of the new method has also been achieved in those cases where bacteria and yeasts resulting from the microbiota of the host (i.e., in the case of vaginal swabs N. 10) were still present. In the mass range 3–6 kDa, the MSP of G3 strain presented peaks of the TYM medium, not detected both in the *Trichomonas* medium N.2 and in the clinical isolates grown in this medium, which were considered relevant by the algorithm of the identification using standard parameter setting (3–15 kDa); the better score obtained with the new range setting could be due to the exclusion of the mass range 3–6 kDa including these peaks.

The reported low sensitivity of MALDI-TOF MS technology [12] is confirmed also in this study where a detection limit of 10^5^ trophozoites/ml was observed. This could not allow the identification of *T. vaginalis* directly in clinical samples, as suggested by the preliminary results obtained for the 3 experimentally seeded samples analysed in this study and for the urine sample N. 20 analysed after only 5 h from its arrival in the laboratory and immediately stored at 4 °C. Moreover, the limited number of reference strains implemented in the database (one strain) could have affected, in these cases, the reliability of the identification by MALDI-TOF MS.

Thus, this technology has a limitation for its potential application to the diagnostic work-flow of trichomoniasis as it can be performed only in samples presenting a very high concentration of the parasite, or after its culture. Anyway, unlike the culture, MALDI-TOF MS does not require the viability of the parasite. Moreover, compared to rapid antigen tests, MALDI-TOF MS is not limited in the application to vaginal secretion, and compared to nucleic acid amplification tests, not widely performed in parasitology laboratories, is less cumbersome and expensive. In any case, the preliminary results obtained in this study could be a starting point for further evolutions of the developed system. The development of an efficient identification method is required to identify protozoa grown on complex liquid media for which interfering substances cannot be completely discarded by the pre-processing protocol before the identification step.

## Conclusions

The importance of this preliminary study lies essentially in the development of a new identification method by MALDI-TOF MS: it has made possible to identify, on the basis of the proteic profile and not on the basis of single markers, a microorganism such as the protozoan *T. vaginalis* which was grown on complex media that could present peaks overlapping those of the microorganism itself in the molecular weight range used for identification purposes by MALDI-TOF MS. In fact, the executive protocol commonly used in diagnostic practice in a range 3–15 kDa was developed for bacteria and fungi which generally require solid growth media not giving interferences in this mass range. Further experiments must be conducted to validate the clinical diagnostic performance of *T. vaginalis* MALDI-TOF MS assay that could be a potential tool for the application also in the diagnostic work-flow in trichomoniasis, as already proposed in recent studies (i.e., [19, 20, 26]) for other parasites.

## Abbreviations

HCCA, α-cyano-4-hydroxy-cinnamic acid; MALDI-TOF MS, matrix-assisted laser desorption/ionization time-of-flight mass spectrometry; MSP, main spectrum profile; PCR, polymerase chain reaction; TYM, trypticase-yeast extract maltose; WHO, World Health Organization

## References

[1] *Trichomonas*. http://www.ncbi.nlm.nih.gov/Taxonomy/Browser/wwwtax.cgi?id=5721. Accessed 14 Aug 2015.

[2] Addis MF, Rappelli P, Delogu G, Carta F, Cappuccinelli P, Fiori PL (1998). Cloning and molecular characterization of a cDNA clone coding for *Trichomonas vaginalis* alpha-actinin and intracellular localization of the protein. Infect Immun.

[3] Hirt RP, Sherrard J (2015). *Trichomonas vaginalis* origins, molecular pathobiology and clinical considerations. Curr Opin Infect Dis.

[4] WHO (2012). Global incidence and prevalence of selected sexually transmitted infections- 2008.

[5] Honigberg BM, Kreier JP (1978). Trichomonads of importance in human medicine. Parasitic protozoa.

[6] Trichomoniasis. http://www.cdc.gov/dpdx/trichomoniasis/. Accessed 14 Aug 2015.

[7] Hegazy MM, El-Tantawy NL, Soliman MM, El-Sadeek ES, El-Nagar HS (2012). Performance of rapid immunochromatographic assay in the diagnosis of *Trichomoniasis vaginalis*. Diagn Microbiol Infect Dis.

[8] Bandea CI, Joseph K, Secor EW, Jones LA, Igietseme JU, Sautter RL (2013). Development of PCR assays for detection of T*richomonas vaginalis* in urine specimens. J Clin Microbiol.

[9] Carlton JM, Hirt RP, Silva JC, Delcher AL, Schatz M, Zhao Q (2007). Draft genome sequence of the sexually transmitted pathogen *Trichomonas vaginalis*. Science.

[10] De Jesus JB, Cuervo P, Junqueira M, Britto C, Silva-Filho FC, Sabóia-Vahia L (2007). Application of two-dimensional electrophoresis and matrix-assisted laser desorption/ionization Time-of-flight mass spectrometry for proteomic analysis of the sexually transmitted parasite *Trichomonas vaginalis*. J Mass Spectrom.

[11] Tran A, Alby K, Kerr A, Jones M, Gilligan PH (2015). Cost savings realized by implementation of routine microbiological identification by Matrix-Assisted Laser Desorption Ionization-Time of Flight (MALDI-TOF) mass spectrometry. J Clin Microbiol.

[12] Croxatto A, Prod’hom G, Greub G (2012). Applications of MALDI-TOF mass spectrometry in clinical diagnostic microbiology. FEMS Microbiol.

[13] Williams TL, Andrzejewski D, Lay JO, Musser SM (2003). Experimental factors affecting the quality and repro-ducibility of MALDI TOF mass spectra obtained from whole bacteria cells. J Am Soc Mass Spectrom.

[14] Calderaro A, Piccolo G, Montecchini S, Buttrini M, Gorrini C, Rossi S (2013). MALDI-TOF MS analysis of human and animal *Brachyspira* species and benefits of database extension. J Proteomics.

[15] Calderaro A, Gorrini C, Piccolo G, Montecchini S, Buttrini M, Rossi S (2014). Identification of *Borrelia* species after creation of an in-house MALDI-TOF MS database. PLoS One.

[16] Calderaro A, Piccolo G, Gorrini C, Montecchini S, Buttrini M, Rossi S (2014). *Leptospira* species and serovars identified by MALDI-TOF mass spectrometry after database implementation. BMC Res Notes.

[17] Calderaro A, Motta F, Montecchini S, Gorrini C, Piccolo G, Piergianni M (2014). Identification of *Dermatophyte* species after implementation of the in-house MALDI-TOF MS database. Int J Mol Sci.

[18] Kassa FA, Shio MT, Bellemare MJ, Faye B, Ndao M, Olivier M (2011). New inflammation-related biomarkers during malaria infection. PLoS One.

[19] Cassagne C, Pratlong F, Jeddi F, Benikhlef R, Aoun K, Normand AC (2014). Identification of *Leishmania* at the species level with matrix-assisted laser desorption ionization time-of-flight mass spectrometry. Clin Microbiol Infect.

[20] Martiny D, Bart A, Vandenberg O, Verhaar N, Wentink-Bonnema E, Moens C (2013). Subtype determination of *Blastocystis* isolates by matrix-assisted laser desorption/ionisation time-of-flight mass spectrometry (MALDI-TOF MS). Eur J Clin Microbiol Infect Dis.

[21] Culha G, Akyar I, Yildiz Zeyrek F, Kurt Ö, Gündüz C, Özensoy Töz S (2014). Leishmaniasis in Turkey: determination of *Leishmania* species by Matrix-Assisted Laser Desorption Ionization Time-Of-Flight Mass Spectrometry (MALDI-TOF MS). Iran J Parasitol.

[22] Magnuson ML, Owens JH, Kelty CA (2000). Characterization of *Cryptosporidium parvum* by matrix-assisted laser desorption ionization-time of flight mass spectrometry. Appl Environ Microbiol.

[23] Moura H, Ospina M, Woolfitt AR, Barr JR, Visvesvara GS (2003). Analysis of four human microsporidian isolates by MALDI-TOF mass spectrometry. J Eukaryot Microbiol.

[24] Villegas EN, Glassmeyer ST, Ware MW, Hayes SL, Schaefer FW (2006). Matrix-assisted laser desorption/ionization time-of-flight mass spectrometry-based analysis of *Giardia lamblia* and *Giardia muris*. J Eukaryot Microbiol.

[25] Wang Y, Cheng Z, Lu X, Tang C (2009). *Echinococcus multilocularis*: proteomic analysis of the protoscoleces by two dimensional electrophoresis and mass spectrometry. Exp Parasitol.

[26] Calderaro A, Piergianni M, Buttrini M, Montecchini S, Piccolo G, Gorrini C (2015). MALDI-TOF mass spectrometry for the detection and differentiation of *Entamoeba histolytica* and *Entamoeba dispar*. PLoS One.

[27] Hirt RP, Noel CJ, Sicheritz-Ponten T, Tachezy J, Fiori PL (2007). *Trichomonas vaginalis* surface proteins: a view from the genome. Trends Parasitol.

[28] Diamond LS (1957). The establishment of various trichomonads of animals and man in axenic cultures. J Parasitol.

